# Metabolic effects of low glycaemic index diets

**DOI:** 10.1186/1475-2891-8-5

**Published:** 2009-01-29

**Authors:** Gabriela Radulian, Emilia Rusu, Andreea Dragomir, Mihaela Posea

**Affiliations:** 1 "Carol Davila" University of Medicine, Bucharest, Romania; 2 "N. Paulescu" National Institute of Diabetes, Nutrition and Metabolic Diseases, Bucharest, Romania; 3 Foundation for Healthy Nutrition, Bucharest, Romania

## Abstract

The persistence of an epidemic of obesity and type 2 diabetes suggests that new nutritional strategies are needed if the epidemic is to be overcome. A promising nutritional approach suggested by this thematic review is metabolic effect of low glycaemic-index diet.

The currently available scientific literature shows that low glycaemic-index diets acutely induce a number of favorable effects, such as a rapid weight loss, decrease of fasting glucose and insulin levels, reduction of circulating triglyceride levels and improvement of blood pressure.

The long-term effect of the combination of these changes is at present not known.

Based on associations between these metabolic parameters and risk of cardiovascular disease, further controlled studies on low-GI diet and metabolic disease are needed.

## Reviews

Obesity is a major global health problem that has been associated with highly occurring disorders such as hypertension, type 2 diabetes, hyperinsulinemia, dyslipidemia, atherosclerosis and certain types of cancer [1,2]. More than one billion adults worldwide are overweight, with ≥ 300 million clinically obese [3]. The epidemic of overweight and obesity, which is rising worldwide, inflicts not only a reduced quality of life and large healthcare-associated costs, but also an increased risk of death [4].

Trend analysis in relation to obesity suggests that in most countries the majority of the population is less active than they should be for maintaining good health, while they are simultaneously eating more than they need [5]. Weight gain is considered as a consequence of excessive energy intake as compared with energy expenditure, while successful weigh loss depends upon achieving negative energy balance [2]. In this context, environmental influences, such as an inactive lifestyle and the consumption of energy-dense diets, appear of overriding importance on excessive weight gain in addition to genetic predisposition [6].

Weight management in the obese may take into account the energy intake and the dietary macronutrient distribution.

A number of nutritional approaches and diets with different proportions of lipids, proteins and carbohydrates are being investigated, which may be energy restricted or prescribed [7,8]. Not only the different macronutrient composition is of interest, but also the micronutrient content and specific dietary components could also be important [9].

The prescription of nutritionally equilibrated low-energy diets is a common strategy for body-weight reduction [10].

Table 1. Influence of the different diets on weight loss [11,12]

**Table 1:** Influence of different diets on weight loss.

**TYPE OF DIET**	**WEEKS**	**WEIGHT LOSS**	**WAIST LOSS**
Low energy diets (800–1200 kcal/zi)	14 weeks	7–13 kg	10 cm waist
Very-low energy diet (400–800 kcal/zi)	2 weeks	4–5 kg	-
High protein diet (30%)	12 weeks	7–9, 5 kg	8–9 cm waist
Low fat diet (20%)	24 weeks	5–6, 5 kg	6–7 cm waist

Low energetic diets can achieve short-term weight loss, but often the slimming process is not sustainable in the long term [13].

The traditional nutritionally adequate low energy diets frequently failed to promote stable weight losses, and the explanations for such limited success were mostly the "poor adherence" to specific low-energy diets.

A study about the effect of energy restriction and diet composition on weight loss and changes in plasma lipids and glucose levels concluded that all energy-restricted diets improve glucose control independently of diet composition, while only the lipoprotein profile was affected by the macronutrient composition [14].

Recently, diets low in carbohydrate (low- carbohydrate diets) have become the focus of international attention since the recent WHO recommendations to reduce the overall consumptions of sugars and some health professionals recommendations to reduce the consumption of rapidly digestible starches that lead to high glucose responses [15].

A study about the influence of low-carbohydrate or low-fat diet on weight loss and risk factors for atherosclerosis in the elderly, obese patients with type 2 diabetes mellitus, shows that the results were more obvious in patients with carbohydrate-restricted diet, with a relative improvement in insulin sensitivity and triglycerides levels [12].

High protein low-carbohydrate diets have been proposed as an alternative to conventional diets, in order to reduce or treat the risk of obesity, CVD and type 2 diabetes mellitus [16].

But this carbohydrate restriction produces a depletion of glycogen stores leading to excretion of bound water, and ketogenic effect.

It has been proposed that the glycaemic index of foods can influence body-weight control [17]. Short-term studies suggest that low-glycaemic index carbohydrates and fiber intake could delay hunger and decrease subsequent energy intake compared with high-glycaemic index foods [18].

The glycaemic index (GI) is defined as "the incremental area under the blood glucose curve following ingestion of a test food, expressed as a percentage of the corresponding area following an equivalent load of a reference carbohydrate, either glucose or white-wheat bread"[19,20].

The GI of a food will vary depending on the rate of digestion. The faster the digestion of a food, the higher is the GI value (>70).

Also, the GI is defined as Food with a low GI (≤ 70) are considered to be favorable in terms of health, particularly for the prevention of obesity, T2DM, and CVD. The GI is affected by a number of factors:

1 the type of carbohydrate (glucose, 138; maltose, 105; saccharose, 75; fructose, 30);

2 the fat and protein content of food (a lower GI is associated with a slowing of gastric emptying);

3 acidity affects gastric emptying and hence the GI a food, the addition of citric acid or other fruits will therefore lower the GI;

4 the physical properties of food (i.e., water content);

5 the presence of viscous soluble fibers; ripeness, cooking, or processing that renders the carbohydrate more digestible (e.g., starch) will increase the GI;

6 the presence of other factors (i.e., insoluble fiber as found in whole intact grains) that slow absorption of the carbohydrate.

After consumption of high GI foods, there is a large, rapid rise in the level of blood glucose, a large insulin response, and glucagons release is strongly inhibited. Low-GI foods may also delay the return of hunger, by slowing gastric emptying. Many low-GI foods are high in fiber content, which prolongs the distension of the gastrointestinal tract, causing increased and prolonged secretion of the gut peptides cholecystokinin, ghrelin, glucagons, glucagons-like-peptid-1, and glucose-dependent insulinotropic polypeptide, all of which have been suggested as potential satiety factors [21,22].

### A. Metabolic effects of low glycaemic-index diets

Observational studies indicate that the GI of the diet may be an important determinant of metabolic risk. The major sources of carbohydrate in the Western diet (highly-refined cereal and potato products) tend to have high GI values, which has been linked to the widespread occurrence of type 2 diabetes and CVD [23].

GI has been shown to be positively associated with the prevalence of the metabolic syndrome and insulin resistance in a cross -sectional study of 2834 subjects from the Framingham Offspring cohort [24]. Odds of having metabolic syndrome were reported to be 41% higher in the highest quintile of dietary GI compared with the lowest quintile (median GI values 84 and 72 respectively), and insulin resistance was found to be increased across quintiles (p < 0.001) [23].

Associations have also been reported between GI and both unfavorable lipid profiles and raised inflammatory status. In 280 women aged 45–70 years from the Nurses Health Study fasting triacylglycerol levels were shown to be positively related to GI [25]. Serum HDL levels have been found to be negatively related to GI in 1420 subjects aged 18–64 years from the 1986–7 Survey of Britain Adults 18–64 years [26], in which GI was the only dietary factor found to be significantly associated with HDL levels in multiple linear-regression analysis. Plasma levels of high-sensitivity C-reactive protein, a sensitive marker of systemic inflammation, were found to be positively associated with both GI and GL in 244 women from the Nurses' Health Study, aged 45–82 years, with a stronger relationship in overweight women than in normal-weight women [27].

**Figure 1:**
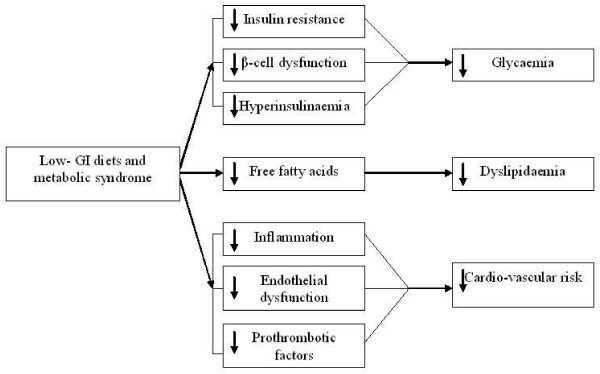
**Low GI diets and metabolic syndrome**. Description: Low GI diets decrease insulin resistance and insulin levels, causing plasma glucose reduction, decrease free fatty acids levels, inflammation and endothelial dysfunction, causing an important reduction of cardiovascular risk factors.

Fig. 1. **Low GI diets and metabolic syndrome**.

Weight loss is an additional potential mechanism by which low-GI diets may contribute to reduced risk of metabolic syndrome.

Induction of a rapid initial weight loss with low-carbohydrates diets may be partly explained by a reduction in overall caloric intake, which may be the result of a great limitation of food choices by the requirements of minimizing carbohydrates intake [28,29], to the initial increase in circulating β-hydroxybutyrate, which may suppress appetite [30] and to the satiating effect of low-carbohydrates diets containing relatively high amounts of protein [31,32]. Some of the initial weight loss may also be explained by a reduction of glycogen stores from liver (5% of liver weight) and muscle (1% of muscle weight). Each gram of glycogen is stored with approximately 3 g of water [33]. Therefore a weight loss of 1–2 kg can theoretically be achieved within the first week of the diet because of substantial glycogen reductions in liver and muscle and excretion of the liberated water in urine [15]. Depending on the rate of glycogen depletion, this process may last up to 7–14 days, after which weight loss slows [34]. It should be noticed in this respect that loss of glycogen and water is not a true measure of weight loss, as their stores will be replenished once the diet is stopped [15].

The rapid large rise in blood glucose following consumption of high-GI food triggers a large insulin response and strongly inhibits glucagons release. For most foods, a good correlation exists between glucose and insulin responses, with high-GI foods eliciting large insulin responses [35], which trigger rapid uptake of nutrients by insulin-responsive tissues and suppress nutrient mobilization. Glucose uptake and glycogen synthesis in skeletal muscle and liver, and lipogenesis in adipose tissue, are increased. Simultaneously, gluconeogenesis and glucose out put by liver and lipolysis are suppressed.

Low-GI diets give a more stable diurnal profile, reducing postprandial hyperglycaemia and hyperinsulinaemia, and attenuating late postprandial rebounds in circulating free fatty acids, all factors that exacerbate various components of the metabolic syndrome [23].

### B. Insulin resistance and insulin secretion

High circulating free fatty acids levels result in lipid accumulation in skeletal muscle and liver, causing insulin resistance in these normally insulin-responsive tissues [36,37], which reduces insulin-stimulated glycogen synthesis in skeletal muscle (the primary pathway for non-oxidative glucose disposal in normal subjects [38] and decrease the ability of insulin to suppress hepatic glucose production and output.

Insulin sensitivity may be negatively affected in the long term as low- carbohydrates, high-fat diets favor an increase of plasma circulating free fatty acids [39,40], which under usual dieting conditions is typically associated with many insulin-resistant states in humans [41,42]. Altered fatty acid metabolism contributes to insulin resistance because of alterations in the partitioning of fat between the adipocytes and muscle or liver [15]. Accumulation of fatty acid and fatty acid metabolites in these insulin-responsive tissues leads to acquired insulin signaling defects and insulin resistance resulting in a reduced glucose transport [43]. The later is thought to results from fatty-acid-induced alterations in upstream insulin signaling events, resulting in decreased GLUT 4 translocation to the plasma membrane. Increased level of intracellular fatty acid metabolites, such as diacylglycerol, fatty acyl CoA's, or ceramides activates a serine/threonine kinase cascade, possibly initiated by protein kinase CӨ. The latter leads to a non-desired phosphorylation of serine/threonine sites on insulin receptor substrates, which then fail to associate with or to activate PI 3-kinase, resulting in decreased activation of glucose transport and other downstream events [15].

**Figure 2:**
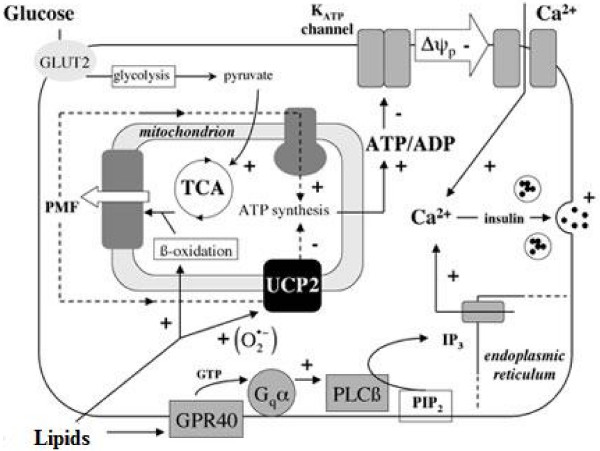
**Regulation of insulin secretion by glucose and lipids**. Description: Insulin secretion is influenced by plasma glucose and lipids that activate on one hand glycolysis and production of pyruvate, and on the other hand β-oxidation and ATP synthesis.

Fig. 2**Regulation of insulin secretion by glucose and lipids **(adapted by Brand et al. (2004). Free Radic. Biol. Med.).

It is observed an inverse relationship between adiponectin and insulinsensivity. Adiponectin is similar in structure to TNF-α (tumor necrosis factor α) which paradoxically appears to be increased in abdominal adipose tissue. Increases in proinflammatory cytokines (interleukine 6, TNF-α, resistin, C- reactiv protein – CRP) reflect overproduction by the expanded adipose tissue mass [44,45]. All of these factors contribute to the exaggerated release of free fatty acids from abdominal adipocytes into the portal system. Free fatty acids have deleterious effects on insulin uptake by the liver and contribute to the increased hepatic gluconeogenesis and hepatic glucose release observed in central obesity [46].

High glucose levels have a glucotoxic effect on β-cells, probably as a result of free radical oxidative damage [47]. Hyperinsulinaemia may reduce β-cell function by causing excess amyloid deposits [48]. High free fatty acids levels lead to triacylglycerol accumulation in β-cells, which reduces insulin secretion [49]. Accordingly, by reducing hyperglycaemia, hyperinsulinemia and free fatty acids levels, low-GI foods may decrease the factors contributing to β-cell failure [23].

### C. Dyslipidaemia

Hyperinsulinaemia and insulin resistance are significantly correlated to dyslipidaemia and contribute to the characteristic alteration of plasma lipid profile associated with obesity.

Low-GI diets may reduce insulin-stimulated activity of 5-hydroxi-3-methylglutaryl-CoA reductase, the rate-limiting enzyme involved in cholesterol synthesis, by reducing insulin levels. Dietary fiber tends to reduce bile acid and cholesterol re-absorption from the ileum, which may inhibit hepatic cholesterol synthesis [47].

Low-carbohydrates diets as well as low-fat diets significantly decreased several biomarkers of inflammation (CRP, TNF-α, IL-6), which play a key role in all stages of the pathogenesis of atherosclerosis [15].

### D. Cardiovascular risk factors

Hyperglicaemia is a continuous risk factor for CVD morbidity and mortality.

The effects of cytokines on peripheral tissues with increased intracellular lipids also lower cellular insulin sensitivity: the surge in lipids promotes proliferation of the vasa vasorum of the arterial media and apoptosis by the medial macrophages with a further release of cytokines. There is an additional complex relationship through an associated pro-inflammatory state and alterations in coagulation. A rise in blood viscosity is induced by the release of profibrinogen and plasminogen activator inhibitor 1 from adipocytes with a fall in plasminogen activator. These changes may explain the role of obesity as a promoter of intracellular inflammatory processes that result in arterial damage. Ethnic differences in CRP may explain some of the variation observed in insulin resistance across populations with comparable weight gain and associated medical complications [46].

A low-GI diet may have beneficial effects on thrombolytic function. The activity of plasminogen activator inhibitor-1, a thrombolytic factor that increases clot and plaque formation, has been found to be 53% lower after 24 days on a low -GI diet compared with a high-GI diet in twenty subjects with type 2 diabetes [50].

Long-term studies are clearly required, as recently, an increased plasma homocysteine level (+6.6%) was observed in individuals that follow strictly a low- carbohydrates diet for several months [51]. In contrast, a low-fat diet induced a decrease of plasma homocysteine by 6–8%. This may be an important observation as the relationship between total plasma homocysteine and CVD is dose dependent and independent of other risk factors. In humans, the effects of homocysteine on endothelial and vascular function and blood coagulation provide explanations for increased CVD risk [15,52,53].

The effects of increased body fatness on cardiovascular function can be predicted. Total body oxygen consumption is increased because of an expanded lean tissue mass and metabolically active adipose tissue, and this is accompanied by an absolute increase in cardiac output. The total blood volume in obesity is increased in proportion to body weight. This increase in blood volume contributes to an increase to the left ventricular pre-load and an increase in resting cardiac output. The increased demand for cardiac output is achieved by an increase in stroke volume: an increase in stroke volume results from an increase in diastolic filling of the left ventricle. This thickening of the wall with dilatation results in eccentric hypertrophy. The cardiovascular adaptation to the increased intravascular volume of obesity may not completely restore normal homodynamic function. Marked systolic dysfunction occurs when the ventricle can no longer adapt to volume overload. Dilatation of the left ventricle cavity radius leads to a decline in ventricular contractility. A combination of systolic and diastolic dysfunction progresses to heart failure [46,54]. Hyperglicaemia exacerbates oxidative stress, which is associated with inflammation, increased blood pressure, accelerated clot formation and decreased endothelium-dependent blood flow [47,55], and which may also worsen insulin resistance [56].

## Conclusion

Long term compliance to a low-GI diet may induce favorable metabolic effects.

A diet high in fruits and vegetables, whole grains and low fat dairy products are important for weight loss.

Reduced hyperinsulinaemia associated with a low-GI diet may reduce CVD risk through effects on oxidative stress, blood pressure, serum lipids, coagulation factors, inflammatory mediators, endothelial function and thrombolytic function [47,49,55-57].

Based on associations between these metabolic parameters and risk of disease, further controlled studies on low-GI diet and metabolic disease are needed [58,59].

Data from long term clinical trials on the metabolic effects on different diets are needed in this area.

## Competing interests

The authors declare that they have no competing interests.
